# A Treatise on Rational Pathology

**Published:** 1853-07

**Authors:** 


*A Treatise on Rational Pathology.* By Dr. J. Henle, Professor of Anatomy and Physiology in Heidelberg. Translated by Henry C. Preston, A. M., M. D. Philadelphia, Lindsay & Blakiston, 1853.

The translator, in his preface to this work, speaks of "the almost entire absence of a complete standard work" on pathology, and trumpets Prof. Henlé to the world as "the *best* modern pathological author." Such parade as this is calculated to awaken large expectations in the profession, which, unless we are greatly mistaken, the book itself will grievously disappoint. It adds nothing to the already brilliant reputation of the Heidelberg professor, but, on the contrary, is likely, we think, to injure his fame.

The first part of the introduction, in which medical systems are discussed, is conceived in lamentably bad taste. Strangely enough, the author manages to lug in religion, to discuss the personality of the devil, to sneer at the clergy, and to have a fling at what he chooses to call the authropomorphism of the Scriptures. Whatever may be thought of his theological views, the absurdity of such a discussion in a work on pathology needs not to be commented upon. With true German dread of the practical, he joins the Oken school in their denunciations of teleological physiology, though he does not tell us whether we are to rest satisfied with visionary omologies, and are to construe all animals into bundles of curiously varied back-bones. On some future occasion we design discussing the comparative merits of the teleological and the homological systems, so that we pass the matter by, for the present. The author then pours his Teutonic ridicule upon what he calls orthodox pathology, and prepares us to expect wonderful revelations from the inner shrine, in the future chapters of his book.

In this we were disappointed. The greater portion of it is taken up with abstract reasonings upon pathological opinions. It reminded us of what Horne Tooke said of Locke, when he called his famous essay on the understanding a treatise on language. Most metaphysicians fall justly under the censure of the acute author of the Diversions of Purley, they discuss *words,* when they think they are profoundly investigating *ideas.* This is very much the case with Henlé in the opening chapters of his book. It is evidently the *words,* disease, health, normality, abnormality, &c., rather than the *ideas* expressed by them on which he wastes so much metaphysical acumen.

The most valuable portion of the work, in our opinion, is that which treats of the various forms of sympathy. This really contains much useful information, and is well reasoned out.

The translator apologizes, and pleads the active duties of his profession as an excuse for the manner in which his work has been done. He also speaks of the difficulty of conveying the ideas of a German metaphysician in any other language than his own. The critic, however, cannot accept any such excuses. Want of time is an excellent apology for not writing a book, but not for writing it carelessly. We wish we could say that these apologies were not required, but, unfortunately, we cannot do so with truth. The translation is, in many places, both obscure and inelegant.

